# A rare but important adverse event associated with adult voluntary medical male circumcision: prolonged bleeding

**DOI:** 10.1186/s12245-015-0056-5

**Published:** 2015-04-10

**Authors:** Moses Galukande, Carol Kahendehe, Eria Buuza, Denis Bbaale Sekavuga

**Affiliations:** Surgery Department, Mulago Hospital, Mulago Hill, P. O. Box 7072, Kampala, Uganda; International Hospital Kampala, Plot 4686 St. Barnabas road, Kisugu, P.O. Box 8177 Kampala, Uganda; Infectious Diseases Institute, Makerere University, Kampala, Uganda

**Keywords:** Prolonged bleeding, Voluntary medical male circumcision, Adverse events, Hemophilia

## Abstract

**Background:**

The majority of bleeding disorders worldwide are undiagnosed. Their moderate or severe forms are associated with considerable morbidity and mortality. In the advent of mass male circumcision for the partial prevention of HIV, undiagnosed and diagnosed cases of bleeding disorders are likely to be increasingly encountered. The ability to screen, diagnose, and manage these cases appropriately will mitigate associated adverse events.

We describe three cases of prolonged bleeding after adult voluntary medical male circumcision (VMMC) and propose program measures.

**Methods:**

A descriptive case series at tertiary urban hospital serving a VMMC program. The cases were recruited consecutively over a 3-year period. Standard laboratory tests were used to confirm diagnosis. Written informed consent was obtained from each subject.

**Results:**

Three cases were described of previously undiagnosed hemophilia A males circumcised during routine VMMC service delivery. They had presented with complaints of prolonged (non-stop or recurring) bleeding.

They were aged 16, 22, and 24 years, of low socioeconomic background, with limited formal education. Whereas two of the three were aware of a tendency to prolonged bleeding from minor cuts, they did not volunteer these histories. The patients were referred to a hospital with the resources to test and administer recombinant factor VIII in Kampala (the capital city) 3, 9, and 16 days after circumcision. Two had received whole blood transfusions enroute to this hospital. All three were diagnosed with mild or moderate hemophilia A. Factor IX levels were all within normal range. In all three cases, the bleeding stopped within hours of the initial factor VIII infusion, and two to three maintenance doses were given over the subsequent 2 to 3 days for each patient.

**Conclusions:**

Sensitization of health workers in safe male circumcision (SMC) programs for pre-operative diagnosis and appropriate referral is highly recommended.

## Background

Close to six million adult voluntary medical male circumcisions have been performed over the past 3 years in 14 sub-Saharan Africa countries for partial prevention of HIV [1]. Apart from achieving the projected numbers, achieving them with minimal adverse events (AEs) is essential. Therefore, identifying AEs, documenting, and sharing these data are important in realizing this goal. One of the commonest AEs after voluntary medical male circumcision (VMMC) is bleeding, which is mostly short lived and normally managed with conservative measures such as applying pressure to the wound and occasionally use of a pressure dressing or suturing. Prolonged bleeding could be due to a bleeding disorder [2]. However, the incidence of post-VMMC bleeding due to bleeding disorders is not known. The purpose of this paper is to describe three cases of a rare but important occurrence of prolonged post-VMMC bleeding.

### Context

These AEs were identified in the last 6 months of a VMMC program that had existed for at least 3 years, during which time approximately 289,000 VMMC were performed in parts of central, northern, and western Uganda by a dedicated team of trained health workers in the Infectious Diseases Institute (IDI)/International Hospital Kampala (IHK) and Baylor-College of Medicine-Uganda, under the auspices of the Ministry of Health, Uganda.

## Methods

This was descriptive; the cases accrued from three VMMC high-volume sites, run by two program implementation partner organizations. The cases were recruited consecutively. Detailed case notes were recorded. On the basis of prolonged bleeding (exceeding 24 to 48 h), laboratory screening and diagnostic tests were done for a coagulation profile, blood count and factors VIII and IX. A standard classification of severity of hemophilia was followed (see Table 1). Case notes and laboratory results were summarized in tables.

**Table 1:** **Classification of hemophilia severity**

**Severity**	**Factor (VIII or IX) level**	**Clinical presentation/treatment**
Mild	5% to 40%	Occasional bleeding, usually only after severe trauma or surgery
Moderate	1% to 5%	Less frequent bleeding which usually follows trauma, surgery, or dental work
Severe	<1%	Factor VIII or IX replacement is needed several times per month for traumatic, or apparently, spontaneous bleeding may be on regular prophylactic factor therapy

## Results

Three cases were described (see Table 2).

**Table 2:** **Summary of case characteristics**

**Age (years)**	**Occupation**	**Residence**	**Delay in days**	**Factor VIII levels (%)**	**Factor IX levels (%)**	**Hb% (g)**	**Blood products**	**Family history**
16	Peasant	Rural	9	4.3	35	7.2	4 WB and 4 FFP units	Positive
22	Fisherman	Rural	16	1.3	69	6.3	2 WB units	Negative
24	Motorcyclist	Urban	3	17	37	10	Nil	Negative

WB, whole blood; FFP, fresh frozen plasma.

### Case presentation 1

A 16-year-old male (primary school dropout) presented with a complaint of continuous bleeding from an incision site for 9 days after VMMC. Prior to referral, he received one unit of whole blood and attempts to achieve homeostasis were made by pressure dressings. The circumcision had been conducted at an IDI field VMMC site a health center IV unit.

#### Past medical/surgical history

The patient reported repeated episodes of spontaneous painful joint swellings since early childhood with subsequent deformity particularly involving the elbow and knee joints.

This patient also had a history of epistaxis with prolonged bleeding; he reported three episodes in the recent past but had not been hospitalized for any. The epistaxis was often preceded by a cold.

#### Family history

He is the third of the four children to both his parents. The first male was deceased; the second is a female who is currently married with children, and the fourth is a male who has never had any bleeding abnormalities. The father is deceased reportedly due to excessive hemoptysis while being treated for pulmonary tuberculosis. The eldest brother died during early childhood at about 5 years due to excessive hemorrhage after a cut wound on his hand. The mother reported that the patient’s paternal great grandfather had bleeding tendencies, which led to his eventual death.

#### On examination

On the first day, the patient was severely pale, with a pulse rate of 122 bpm, blood pressure 120/78 mmHg, and was oozing from three points at the circumcision site. The initial laboratory investigations indicated: anemia with Hb 7.2 g/dl and Hct 23%; INR 1.01, blood group O+, APTT 67 s, bleeding time 2 min, 10 s, clotting time was 3 min. Factor VIII measurement was at 4.3% (below the normal range, see Table 1). Factor IX was normal within the normal range 35%.

#### Interventions

Interventions prior to factor VIII infusion included pressure dressing and cauterization. These did control the bleeding but for only 24 h. Four units of fresh whole blood, four of fresh frozen plasma (FFP), and vitamin K injection of 10 mg were also given. Factor VIII test results returned on the seventh day of admission showing low levels. Recombinant factor VIII was administered over 2 days, and the bleeding stopped.

Telephone follow-up at 6 weeks revealed that he was well. The patient and his family were educated about hemophilia A and given information leaflets.

### Case presentation 2

A 22-year-old fisherman from rural northern Uganda presented with a complaint of continuous bleeding 16 days after circumcision. Prior to presentation to this facility, he received two units of whole blood transfusion from a health center IV, a field VMMC post managed by the Baylor Uganda program. He was transported to Kampala a distance of over 500 km. He reported a past history of prolonged bleeding of up to 3 days after minor cuts since the age of 7 years.

#### Family history

There was no known family member or male siblings with bleeding disorders though two siblings died in infancy due to unspecified febrile illnesses.

#### On examination

He had severe pallor of the mucous membranes, was not jaundiced, was afebrile, and in good general physical state. Vitals signs were unremarkable apart from tachycardia of 100 bpm and BP 100/60 mmHg.

#### Laboratory tests

The patient had a low Hb of 6.3 g, with a normal platelet count of 313 × 10^9^/l (150 to 450), and APTT 121 (36 to 40 s). He had low factor VIII levels, 1.3% which is categorized as moderate hemophilia A. Factor IX levels were normal was 69% (70 to 140).

#### Intervention

He received recombinant factor VIII within 24 h of admission and two other loading doses. The bleeding stopped, and he was discharged on the third day after hospital admission.

Telephone follow-up at 6 weeks revealed that the patient was doing well.

### Case presentation 3

A 24-year-old boda-boda cyclist (a commercial commuter motor cyclist) presented with a complaint of non-stop bleeding, 3 days after circumcision at an IDI/IHK urban VMMC site in Kampala. He had returned to the site thrice for pressure dressing, exploration, and suturing but without much success in stopping the bleeding. He had a history of prolonged bleeding from minor cuts before but had not comprehended its significance. There was no similar history from his siblings or parents.

#### On examination

He was in good general condition, with mild-moderate pallor, a pulse of 64, blood pressure 103/69 mmHg, and was afebrile. A pressure bandage over the circumcision wound was blood-soaked.

#### Investigations

The patient’s Hb was low 10 g%, with low factor VIII levels of 17%. The factor IX levels were normal and APPT 47 (26 to 35).

#### Treatment

Treatment consisted of 1,000 IU factor VIII, followed by three other doses on separate days.

Uganda has a Haemophilia Foundation, which encourages registration of patients with bleeding disorders with the foundation. They provide support in terms of education and supply of factor replacements among other functions. All the three cases were registered with the foundation as members at a fee of $15 per annum.

## Discussion

It is estimated that only 30% of the men and women with bleeding disorders are presently diagnosed worldwide [3]. Mild and moderate forms of hemophilia may not be diagnosed until a first medical or dental procedure, and for some of these males, circumcision is the first procedure performed. In the advent of VMMC for partial HIV prevention, we anticipate that many men with varying severity of bleeding disorders will be found. Those with moderate to severe forms may be diagnosed, and those with milder forms may be missed.

In this paper, three cases were diagnosed out of approximately 289,000 circumcisions performed at designated sites, giving a rate of approximately 1 in 96,000 procedures. The estimated incidence of hemophilia, a more common form of bleeding disorders, is estimated at 6.6 ± 48 per 100,000 in lower income countries and higher in high-income countries at 12.8 ± 6.0 (mean ± SD) per 100,000 births [4]. Theoretically, therefore, out of six million circumcisions done a few hundred should have been encountered, even though some die before reaching the program eligible age for circumcision (10 to 12 years).

We need to heighten the level of suspicion for those who experience prolonged bleeding after minor trauma in their past medical and family histories. To extract these histories may require non-casual probing. Possibly in those suspected cases, circumcision should not be performed in their brothers or maternal cousins until there is assurance that no bleeding dyscrasia exists.

The two main types of hemophilia - hemophilia A (due to factor VIII deficiency) and hemophilia B (due to factor IX deficiency) - exist. They are clinically almost identical and may be associated with spontaneous bleeding into joints and muscles and internal or external bleeding after injury or surgery [5]. In this case series, we encountered only type A.

Hemophilia affects people of all races and ethnic origins globally. The conditions are both X linked, and virtually, all sufferers are males; however, female carriers may also bleed abnormally [5]. Approximately one third of patients with hemophilia have no known family history of the disease as with cases 2 and 3. This could be either because of new genetic mutations or because previous affected generations either had daughters who are only carriers or sons who died early in childhood from undiagnosed hemophilia or any other causes or where not symptomatic [5].

Circumcising patients with bleeding disorders is possible but requires resources and preparations. Giving systemic prophylaxis for factor substitution, desmopressin acetate, and using diathermic knives designed for bloodless circumcision have been described. Fibrin glue is also described as effective in mitigating bleeding [6,7].

Excluding patients with bleeding disorders from safe male circumcision (SMC) may have negative psychosocial effects including stigmatization for those perceiving male circumcision as mandatory for cultural or religious reasons [6,7]. For those diagnosed prior to safe male circumcision, they should be referred to centers that are equipped to deal with such.

### Limitation

Mild forms of bleeding disorders may only be reported as ‘mild’ AEs to or by the respective VMMC sites and therefore missed out altogether. A high level of suspicion is needed to pick these up.

## Conclusions

VMMC programs should emphasize pre-circumcision screening by conducting thorough family and medical histories. Any case of suspected prolonged bleeding perhaps equal or exceeding 24 h should be tested. Establishing clear pathways for referrals of suspected cases to facilities with the capacity to confirm diagnosis and provide factor concentrates and other resources to minimize AEs is recommended. Surveillance mechanisms should be strengthened to detect these occurrences more.

### Consent

Written informed consent was obtained from the patients for the publication of this report.

## Abbreviations

IV: intravenous
MC: male circumcision
SMC: safe male circumcision
VMMC: voluntary medical male circumcision
